# Psychometric Properties of the Self-Efficacy Scale among Undergraduate Students in Malaysia

**DOI:** 10.21315/mjms2019.26.3.10

**Published:** 2019-06-28

**Authors:** Liu Kien Ting, Garry Kuan, Wan Nor Arifin, Kueh Yee Cheng

**Affiliations:** 1 Unit of Biostatistics and Research Methodology, School of Medical Sciences, Universiti Sains Malaysia, Kubang Kerian, Kelantan, Malaysia; 2 Exercise and Sports Science Programme, School of Health Science, Universiti Sains Malaysia, Kubang Kerian, Kelantan, Malaysia

**Keywords:** composite reliability, factor analysis, internal feeling, competing demands, situational, confirmatory factor analysis

## Abstract

**Background:**

Self-efficacy (SE) is a person’s belief in his or her own capability to perform and accomplish a task that could produce a favourable outcome, despite facing obstacles. This study aimed to confirm the validity and reliability of an SE scale among undergraduate students at the Health Campus of the Universiti Sains Malaysia.

**Methods:**

A cross-sectional study was conducted among the undergraduate students using a self-administered questionnaire. After using a purposive sampling method, 562 students completed the questionnaire. Mplus 8 was employed to conduct the confirmatory factor analysis on the psychometric properties of Bandura’s 18-item SE scale with three factors (internal feeling, competing demands and situational). Then, the composite reliability was calculated for each factor.

**Results:**

Most of the students were Malay (73.3%) females (79.0%) who exercised 2.62 times a week for an average of 43.37 min per session. The final measurement model was obtained after removing six problematic items, and the model was deemed fit based on several indices [Root Mean Square Error of Approximation (RMSEA) = 0.067, Standardised Root Mean Square Residual (SRMR) = 0.004, Comparative Fit Index (CFI) = 0.924]. The composite reliability values of the three factors were acceptable (0.65 to 0.84).

**Conclusion:**

The simplified 12-item SE scale with three factors displayed good fit indices with regard to the data, and they were considered to be acceptable for the current sample.

## Introduction

Self-efficacy (SE) is a person’s belief in his or her own capability to perform and accomplish tasks that produce favourable outcomes, despite facing obstacles. SE plays an essential role in assisting individuals with high SEs to perform tasks well after they have experienced more positive emotions (1). SE is the key construct in social cognitive theory that is used to elucidate the factors affecting exercise behaviour among older individuals (1–4).

According to Bandura’s social cognitive theory, SE represents the psychological construct (i.e. the mediator) linked to a wide range of healthy behaviours (4). SE has been proven to be an important determinant that influences an individual’s performance based on the type of activity, the effort committed to the activity and the capability to overcome pressure when faced with obstacles (3). Thus, more successful people tend to set challenging goals and maintain stronger commitments toward reaching them, and they tend to avoid the failures from their previous experiences, which suggests that they have learned from their past experiences. Moreover, they treat every failure as a learning opportunity and as motivation to reach optimum competency using a different approach or strategy.

In contrast, people who doubt their own abilities to accomplish difficult tasks often view failure as a threat (5). They tend to avoid difficult tasks based on their weakness or the obstacles that have prevented them from becoming successful. In addition, they tend to give up quickly when faced with difficulty or failure, and they tend to easily lose faith in their capabilities. Many studies have suggested that various affective symptoms can negatively influence exercise SE, such as depression (6–8) and anxiety (9). Moreover, the factors that have been related to decreased physical activity (PA) include medical problems, negative experiences, a fear of activity related experiences, having a past history of a sedentary lifestyle, an insufficient understanding of the benefits of PA, living in an unsafe neighbourhood and a lack of companionship (10, 11).

Efforts have been made by various researchers to measure an individual’s SE level with regard to adhering to PA when faced with exercise challenges. For example, the SE scale developed by Bandura (4) has been used to determine an individual’s belief and effort when engaging in PA. That SE scale was first applied to English communities (4); then, the scale was translated into Korean (12), Persian (13) and Dutch (14). Later, it was employed among Korean, Iranian and Dutch populations to evaluate its reliability and validity. However, we found no such scales that were available for use in the Malaysian population.

Many studies have reported that SE is an important element for assessing the behavioural intention in addition to actual behaviour (12, 15– 17). Some previous studies have shown that the perceived SE could lead to a behavioural change (18). According to Kim (19), those students with high SEs and confidence levels were more likely to engage in PA, despite facing obstacles. They were expected to better endure initiating and maintaining PA than the individuals with lower SEs. One study conducted among university students in Malaysia indicated that SE was associated with an individual’s behavioural changes related to exercise (20). Although there are many validated instruments available to measure SE, no properly validated instruments are available for measuring SE among different Malaysian populations. Therefore, this study was designed to determine the validity and reliability of an SE scale related to PA among undergraduate students in Malaysia using a confirmatory factor analysis (CFA).

## Materials and Methods

### Study Design and Procedures

A cross-sectional study was conducted in one single period of time at Health Campus, Universiti Sains Malaysia (USM) from 29 October 2017 to 30 April 2018. In the current study, data were collected through a selfadministered questionnaire and were distributed to the students after their co-curriculum classes. Participants were briefed about the research purpose and those who are interested filled the questionnaires. The volunteers took approximate 10 min to 15 min to complete the questionnaire.

### Participants

Undergraduate students from the Health Campus, USM were recruited as the study participants. A purposive sampling method was used to recruit participants who participated in co-curricular from sports, art and uniform clusters. This sampling method was used to ensure that a wide variety of responses among students from sports, art and uniform clusters and the scores were represented by particular characteristics of the students. Thus, this could reduce the floor and ceiling effects of participants’ response on the scale. Since co-curricular is a compulsory unit for all undergraduate students, thus students from first to final years of studies were recruited in this study.

### Instruments

The quantitative data were gathered with two sections, the demographic information and the SE scale. Demographic information such as age (years), gender, ethnicity, course and years of study, exercise frequency (per week), and exercise period per session (min) were included.

The SE scale which comprised of 18 items and three factors, was developed by Bandura (4). A psychometric study conducted by Shin et al. (12) among the Korean students showed three factors of SE: internal feelings (seven items), competing demands (five items), and situational (six items). By using a 5-point Likert-type scale, participants were required to choose their answer from ‘1 = cannot do’, to intermediate degrees of confidence ‘3 = moderately certain can do’, to complete confidence ‘5 = certain can do’. The participants rated ‘confidence’ indicated they were confident to engage in regular physical activity (three or more times a week) under the various circumstances (e.g., “when I am feeling tired”). The internal consistency of SE scale was 0.89 (4). The two weeks test-retest reliability of SE scale was 0.86 (4).

### Data Analysis

For the data analysis, the Mplus version 8 for confirmatory factor analysis (CFA) was used. The level of statistical significance was set at *P* < 0.05. Participant’s characteristics were presented by descriptive information. The continuous variables were presented as mean and standard deviation (SD); meanwhile, categorical variables were presented in frequencies (*n*) and percentages (%).

In CFA, several fit indices were used to validate the factor structure of the SE scale. The fit indices were Standardised Root Mean Square Residual (SRMR), Root Mean Square Error of Approximation (RMSEA), Comparative Fit Index (CFI) and Tucker-Lewis Index (TLI).

## Confirmatory Factor Analysis

The robust multiple linear (ML) estimator was used in the analysis. Multiple linear regression (MLR) is the maximum likelihood parameter estimates with robust standard errors (21). The present study employed guidelines as recommended by Hair et al. (22) throughout analysing the factor structure of CFA. To determine the factor structure of the CFA model, the characteristics of different fit index guidelines were used. The recommended cut of value for CFI and TLI are more than 0.92, SRMR less than 0.08 and RMSEA less than 0.07.

The modification was made by adding error covariance based on modification index (MI) value of more than 10, and standardised factor loading of more than 0.4. According to DeVon et al. (23), the standardised factor loading more than 0.4 still is considered as acceptable in the validation study.

Measurement model was re-specified after adequate theoretical support was carried out by the authors. Model re-specification included identified and removing problematic items, and adding error covariance among items within the same factor. Items with the lowest standardised factor loading were removed once at a time and re-analysed until model fit indices met the recommended guideline.

Correlation among factors was examined to achieve discriminant validity (*r* ≤ 0.85). This was to ensure that no high correlation between two different factors (24) that could cause the model to have poor discriminant validity.

## Results

### Socio-Demographic Characteristics

Five hundred and sixty-two students with the mean age of 19.81 (1.22) ranging from 17 to 27 years old were recruited in this study. Among all the university students, female students comprised of 444 (79%) while male students comprised of 118 (21%). In term of exercise frequency, the average of students’ involvement in physical activity was approximately 43 min per week and three times per week.

### Descriptive Statistics

The 18 questions in the SE model are shown in Table 1. The students’ SE responses are displayed in frequency and percentage (%). From the descriptive results, it showed that most of the students were moderately confident in all situations, whereas the minority chooses completely confident in their preferences. Among all questions, 239 students (42.5%) indicate moderately confident as their choice in SE15 (without support from my family and friends). While 17 students (3.0%) were completely confident that they will not stick to exercise routine when they are feeling tired (SE1). In SE, there are seven items for factor internal feelings, thus the lowest and highest scores were seven and 35, respectively. There were 0.2% (*n* = 1) participants scored the lowest score of seven and none of the participants scored the highest score of 35. For five items in factor competing demands, there was 0.2% (*n* = 1) participants had the lowest and highest score, five and 25. There were small floor effect, 1.1% (*n* = 6) and ceiling effect 0.4 percent, (*n* = 2) in factor situational with six items. Hence, there was no floor and ceiling effect found in SE.

## Confirmatory Factor Analysis

CFA was used to assess the data to determine how well the observed variables fit the latent variables. The initial hypotheses measurement model (Model 1) consists of three latent variables (subscales) with 18 observed variables (items) were investigated to identify problematic items. The model fit indices for seven models is shown in Table 2. The results showed a poor fit of data in Model 1, Table 2 with CFI = 0.657, TLI = 0.603, SRMR = 0.149, RMSEA (90%CI) = 0.110 (0.103, 0.116), RMSEA *P*-value < 0.001.

Factor loadings of Model 1 to Model 6 are presented in Table 3. From an examination of CFA results in Model 2, item SE3 “During bad weather” which had the lowest factor loading with 0.111 was removed. The model was respecified and fit indices were re-examined under Model 2 in Table 2. Although there is a slight improvement in fit indices, yet Model 3 did not show the good fit of the model. Item SE9 “When I feel physical discomfort when I exercise” with the lowest factor loading, 0.233 was removed after considering expert’s opinion. Model 4 showed the overall poor fit of data with a factor loading of item SE1 (0.213) “When I am feeling tired” was removed. Since fit indices still not considered as sufficient, further examination was carried out.

Item SE15 “without support from my family and friends” with lowest factor loading, 0.233 was removed in Model 5. Although the fit indices improved drastically, they still did not represent a good fit of the model to data. Further examination was carried out in Model 6 and item SE14 “If I don’t reach my exercise goals” was identified as a problematic item. After contemplating the theoretical consideration, item SE14 was removed with low factor loading of 0.228.

Model 6 with poor fit indices suggested item SE10 to be removed from the data; however, expert recommended SE10 “After a vacation” to be retained in the model. Item SE18 “After experiencing family problems” was suggested to be less important based on expert’s opinion and considering low factor loading (0.385). Thus, the final model (Model 7) was established with six items removed (SE3, SE9, SE1, SE15, SE14, and SE18) and displayed good fit indices to the data (Table 4).

Based on the final model, composite reliability (CR) was computed and presented in Table 4. CR value of three factors was ranged from 0.652 to 0.841, which indicated moderate to good.

The factors correlations between competing demands and internal feelings as well as situational and competing demands revealed a statistically significant (*P* < 0.001), positive correlation, *r* = 0.250 and *r* = 0.599, respectively. The results of both pairs showed little correlation and moderate to good correlation. The factor correlation between situational and internal feelings showed a non-significant (*P* = 0.258) positive and weak correlation (*r* = 0.066) among undergraduate students. Since the *r* value is less than 0.85, the discriminant validity among the three factors was achieved.

## Discussion

The present study showed the psychometric properties of an SE scale among undergraduate students at the Health Campus of the USM using a CFA. The findings showed that Model 7 in Table 2 exhibited the most satisfactory values for the CFI and TLI after several trials. However, the six items that were found to be problematic were removed iteratively due to low standardised factor loadings or because they caused a reduction in the goodness-of-fit model. Therefore, the original 18-item SE model was reduced to 12 items.

Some of the problematic items that caused the model to be unstable were omitted because the meaning of the content was questioned by the participants. These problematic items were: SE 3: “During bad weather”, SE 9: “When I feel physical discomfort when I exercise” and SE 1: “When I am feeling tired”. The content of these items was confusing, and each participant’s understanding was different. In addition, after considering the expert’s opinion and reviewing the meaning of the items, the following items were omitted based on low standardised factor loadings: SE 15: “Without support from my family and friends”, SE 14: “If I don’t reach my exercise goals” and SE 18: “After experiencing family problems”.

The 12-item simplified version of the scale was in agreement with the results of studies from several countries, including Korea, Iran and the Netherlands. Although the factor loading of SE10 was less than 0.4, and it indirectly affected the composite reliability, the item was considered to be important after obtaining the expert’s opinion. Since SE10 did not affect the overall model fitness, the item was included in the study. Among all studies which adapted the SE scale from Shin, Jang and Pender (12) demonstrated the 18 items with three latent variables were not stable among different study populations. For instance, the researchers that translated the English version of the SE scale to other languages, like Persian (13) and Dutch (14), reported that the original 18 items were reduced to 17 and 13, respectively, due to low standardised factor loadings. However, the original English version of the SE scale was utilised among Australian cardiac patients by Everett et al. (25), who reported high internal consistency (α = 0.95) and demonstrated no floor or ceiling effects. Moreover, one SE scale translated to another language, Arabic (26), also showed good internal consistency (α = 0.89).

For the discriminant validity, three pairs of factors (internal feelings, competing demands and situational) in this study showed no intercorrelations with the other factors. According to one study (24), the occurrence of multiple factors in the CFA model caused the model to have poor discriminant validity. Thus, this study showed that there was strong evidence that each factor was represented by its item.

There are several limitations to be acknowledged in this study. Since the present study collected data from a university, it limits the generalisability to different tertiary educational settings. Thus, the results of this study cannot be generalised across other universities, and they may not represent all university students in Malaysia. Moreover, this study only employed quantitative methods to examine the students’ PA behaviours. Selfadministered methods may create some biases due to dishonest and insincere responses from the study participants. As a result, the reliability of the scale may be a concern. In order to handle this matter, each student was encouraged to answer the questions honestly, based on their point of view, and to avoid discussing the questions with their friends. Finally, the sample was comprised of a majority of female students, which was consistent with other studies conducted at universities in Malaysia (27, 28). Thus, further validity testing, such as an invariance test between the genders, could not be conducted using the present sample. Future research should examine the measurement invariance of the SE scale to confirm that the measure has the same meaning across both genders.

## Conclusion

The simplified 12-item SE scale with good psychometric properties was found to be fit and acceptable among the university students. This simplified version of the 18-item SE scale could be used to assess the confidence level of an individual to initiate the intention to exercise. Moreover, the SE scale could be the best predictor for individuals to initiate or maintain exercise, despite facing obstacles. Individuals with high SEs have stronger beliefs that encourage them to engage in intended behaviours. However, future studies should be conducted among students from other universities in order to test the psychometric properties of this SE scale. In addition, this simplified SE scale could be made more useful by adding confounding variables, such as gender (e.g. male and female), educational level (e.g. undergraduate and postgraduate) and curriculum involvement (e.g. sports group, art groups and uniform groups).

## Figures and Tables

**Table 1:** Summary of item descriptive for SE model, *n* = 562

	Score				
Items	Not at all confident *n* (%)	Somewhat confident *n* (%)	Moderately confident *n* (%)	Very confident *n* (%)	Completely confident *n* (%)
**SE1**	132 (23.5)	173 (30.8)	201 (35.8)	39 (6.9)	17 (3.0)
**SE2**	62 (11.0)	152 (27.0)	225 (40.0)	87 (15.5)	36 (6.4)
**SE3**	159 (28.3)	154 (27.4)	167 (29.7)	56 (10.0)	26 (4.6)
**SE4**	122 (21.7)	155 (27.6)	177 (31.5)	79 (14.1)	29 (5.2)
**SE5**	51 (9.1)	134 (23.8)	217 (38.6)	118 (21.0)	42 (7.5)
**SE6**	51 (9.1)	144 (25.6)	177 (31.5)	130 (23.1)	60 (10.7)
**SE7**	56 (10.0)	145 (25.8)	209 (37.2)	104 (18.5)	48 (8.5)
**SE8**	80 (14.2)	159 (28.3)	187 (33.3)	99 (17.6)	37 (6.6)
**SE9**	86 (15.3)	172 (30.6)	218 (38.8)	65 (11.6)	21 (3.7)
**SE10**	49 (8.7)	123 (21.9)	189 (33.6)	146 (26.0)	55 (9.8)
**SE11**	104 (18.5)	142 (25.3)	189 (33.6)	94 (16.7)	33 (5.9)
**SE12**	133 (23.7)	142 (25.3)	163 (29.0)	102 (18.1)	22 (3.9)
**SE13**	68 (12.1)	152 (27.0)	183 (32.6)	115 (20.5)	44 (7.8)
**SE14**	42 (7.5)	104 (18.5)	227 (40.4)	136 (24.2)	53 (9.4)
**SE15**	92 (16.4)	141 (25.1)	239 (42.5)	65 (11.6)	25 (4.4)
**SE16**	132 (23.5)	165 (29.4)	137 (24.4)	86 (15.3)	42 (7.5)
**SE17**	95 (16.9)	161 (28.6)	187 (33.3)	86 (15.3)	33 (5.9)
**SE18**	90 (16.0)	181 (32.2)	183 (32.6)	78 (13.9)	30 (5.3)

Notes: Items on SE model

**Table 2:** Model fit indices for seven models of SE

Model	CFI	TLI	SRMR	RMSEA (90%CI)	RMSEA *P*-value
Model 1 (Initial)	0.657	0.603	0.149	0.110 (0.103, 0.116)	< 0.001
Model 2^a^	0.724	0.676	0.128	0.100 (0.093, 0.106)	< 0.001
Model 3^b^	0.759	0.714	0.118	0.097 (0.090, 0.105)	< 0.001
Model 4^c^	0.838	0.804	0.091	0.082 (0.074, 0.090)	< 0.001
Model 5^d^	0.857	0.825	0.091	0.082 (0.073, 0.090)	< 0.001
Model 6^e^	0.890	0.861	0.082	0.077 (0.067, 0.086)	< 0.001
Model 7^f^	0.924	0.902	0.064	0.067 (0.057, 0.078)	0.004

CFI = Comparative Fit Index, TLI = Tucker-Lewis Index, SRMR = Standardised Root Mean square Residual, RMSEA = Root Mean Square Error of Approximation, CI = Confidence Interval, Cl fit = Close fit

a Measurement model with SE3 was removed

b Measurement model with SE3 and SE9 were removed

c Measurement model with SE3, SE9, and SE1 were removed

d Measurement model with SE3, SE9, SE1, and SE15 were removed

e Measurement model with SE3, SE9, SE1, SE15, and SE14 were removed

f Measurement model with SE3, SE9, SE1, SE15, SE14, and SE18 were removed

**Table 3:** Standardised factor loading for six models of SE model

Model	Model 1 (Initial)	Model 2	Model 3	Model 4	Model 5	Model 6
Factors and items	Standardised factor loadings					
**Internal feelings**						
SE1	0.254	0.237	0.213	-	-	-
SE2	0.576	0.570	0.561	0.547	0.547	0.546
SE3	0.111	-	-	-	-	-
SE5	0.734	0.733	0.730	0.728	0.728	0.727
SE6	0.866	0.877	0.893	0.909	0.910	0.911
SE7	0.799	0.797	0.793	0.784	0.784	0.783
SE9	0.246	0.233	-	-	-	-
**Competing demands**						
SE4	0.717	0.718	0.721	0.721	0.741	0.763
SE8	0.724	0.724	0.724	0.724	0.740	0.735
SE10	0.356	0.356	0.354	0.354	0.334	0.316
SE14	0.263	0.262	0.259	0.258	0.228	-
SE15	0.236	0.235	0.234	0.233	-	-
**Situational**						
SE11	0.686	0.686	0.685	0.685	0.686	0.686
SE12	0.726	0.726	0.727	0.727	0.729	0.730
SE13	0.647	0.648	0.648	0.648	0.648	0.647
SE16	0.634	0.634	0.634	0.635	0.633	0.633
SE17	0.674	0.674	0.674	0.674	0.674	0.675
SE18	0.393	0.392	0.391	0.389	0.386	0.385

**Table 4:** Standardised factor loading and composite reliability for Model 7

Model	Model 7	Composite reliability (95%CI)
Factors and items	Standardised factor loading	
**Internal feelings**		
SE1	-	0.841 (0.816–0.865)
SE2	0.545	
SE3	-	
SE5	0.727	
SE6	0.913	
SE7	0.782	
SE9	-	
**Competing demands**		
SE4	0.762	0.652 (0.600–0.703)
SE8	0.735	
SE10	0.317	
SE14	-	
SE15	-	
**Situational**		
SE11	0.684	0.806 (0.779, 0.834)
SE12	0.736	
SE13	0.654	
SE16	0.633	
SE17	0.665	
SE18	-	
